# Acute otitis media diagnosis in childhood: still a problem in 2023?

**DOI:** 10.1186/s13052-024-01588-y

**Published:** 2024-01-25

**Authors:** Francesco Folino, Marco Caruso, Pietro Bosi, Mirko Aldè, Sara Torretta, Paola Marchisio

**Affiliations:** 1 Fondazione IRCCS Cà Granda Ospedale Maggiore Policlinico di Milano, Via F. Sforza 35, 20122 Milano, Italy; 2 Department of Clinical Sciences and Community Health, University of Milan, Milan, Italy; 3 Department of Pathophysiology and Transplantation, University of Milan, Milan, Italy

**Keywords:** Otitis media, Children, Otoscopy, Middle ear, Infection

## Abstract

**Background:**

Diagnosis of acute otitis media (AOM) in children can be challenging, given that symptoms are often non-specific or absent, and that the direct observation of the tympanic membrane in its entirety through otoscopy can sometimes be difficult. The aim of this study is to assess the diagnostic concordance in detection of AOM episodes between primary care paediatricians and physicians especially trained in paediatric otoscopy, and to characterize the most misleading elements in diagnostic failure.

**Methods:**

Consecutive clinical charts of children regularly followed for recurrent AOM (RAOM, i.e.: >3 episodes in 6 months or > 4 episodes in 1 year) at our Otitis Media paediatric outpatient clinic were retrospectively screened, in order to collect any diagnosis of AOM episode (and the related clinical findings/middle ear complaints) performed by primary care paediatricians/emergency room paediatricians. Diagnosis of AOM episode was validated by the same experienced physician (FF) in case of otoscopic relief of a bulging eardrum with at least one of the following: hyperaemia or yellow-like colour. The diagnostic concordance in detection of AOM episodes between primary care/emergency room paediatricians and our internal validator was expressed as the percentage of matching diagnosis.

**Results:**

One hundred and thirty-four single AOM episodes occurring in 87 children (mean age: 26.9 +/- 18.9 months) were included in the analysis. Diagnostic concordance in detection of AOM episodes between primary care/emergency room paediatricians and our internal validator was reported in 72.4% of cases. The most common pitfall found in our study was the misleading diagnosis of AOM in case of hyperaemic tympanic membrane without bulging (32/37 out of non-validated diagnoses).

**Conclusions:**

AOM diagnosis still represents a relevant issue among paediatricians in our country, and the presence of tympanic membrane hyperaemia without concomitant bulging can be confusing.

**Supplementary Information:**

The online version contains supplementary material available at 10.1186/s13052-024-01588-y.

## Background

Acute otitis media (AOM) is defined as the presence of middle-ear effusion (MEE) in combination with the acute onset of one or more signs of inflammation of the middle ear [1]. It is one of the most frequent paediatric diseases: in Europe, the incidence of AOM in outpatient care is 256 episodes per 1000 people-year, with a peak of 22.2 episodes per 100 children in Italy [2, 3]. Approximately 2 ⁄ 3 of children under 3 years of age have had at least one AOM episode, and about 1⁄ 3 has recurrences (Recurrent acute otitis media or RAOM, defined as > 3 episodes in 6 months or > 4 episodes in 1 year) [4].

Being one of the most common childhood diseases, AOM represents the most common cause of antibiotic prescription in pediatrics [5] and poses a significant economic burden: an investigation conducted in seven different European countries estimated a total cost per AOM episode ranging from 332.00 euros in the Netherlands to 752.49 euros in the United Kingdom [6].

Performing a correct diagnosis is therefore fundamental in order to reduce costs and to avoid unnecessary resort to antibiotic therapies. Despite this, the international literature reports the high number and the considerable clinical relevance of AOM misdiagnosis [7, 8].

AOM symptoms are often non-specific or absent [3], hence the direct observation of the tympanic membrane (TM) through otoscopy represents the central step for a correct diagnosis. Nevertheless, AOM identification can be challenging, especially in the paediatric age, also considering that the setting in which it should be performed is not always ideal: the child could be not compliant, the tools inadequate and the TM could be visualized only partially [9].

The aim of this study is to assess the diagnostic concordance in detection of AOM episodes between primary care paediatricians and physicians especially trained in paediatric otoscopy, and to characterize the most misleading elements in diagnostic failure.

## Methods

### Study design and setting

A retrospective review of clinical charts related to children prospectively recruited between 1st January 2015 to 31st December 2021 and regularly followed for RAOM in the Otitis Media paediatric outpatient clinic of Fondazione IRCCS Ca’ Granda, Ospedale Maggiore Policlinico (Milan, Italy) for recurrent or chronic middle ear infections.

The study belongs to a larger project aimed at retrospectively study clinical charts of children regularly followed for recurrent or chronic middle ear infections; the protocol was approved by our local Ethics Committee of Fondazione IRCCS Ca’ Granda, Ospedale Maggiore Policlinico (Milan, Italy), and it was conducted in accordance with the principles of good clinical practice.

### Interventions

Consecutive clinical charts were screened in order to record any previous diagnosis of AOM episode performed by primary care paediatricians/emergency room paediatricians, and the reported otological signs of middle ear infections and related clinical findings.

For each episode of middle ear complaint, we considered the acute onset of potential symptoms associated with AOM (fever and ear pain) and the described characteristics of the tympanic membrane (TM), most importantly position (normal/retracted/bulging) and whether it appeared hyperaemic or not; we further defined if the conclusive diagnosis of AOM was confirmed or not by our internal validator, according to the description provided by the physician. Diagnosis of AOM episode was validated by the same experienced physician (FF) in case of otoscopic relief of a bulging eardrum with at least one of the following associated characteristics: hyperaemia or yellow-like color [1].

Single episodes with a final diagnosis of AOM were included in the analysis only when an accurate description of the tympanic membrane was available, performed by a primary care paediatrician or an emergency room paediatrician. Episodes characterized by ear discharge were excluded, as this element represents a feature of certain diagnosis. In the case of incomplete or missing data, the chart was excluded from analysis.

### Statistical analysis

Features of each episode were transcribed on an Excel electronic database (Excel 2016 v16.0, Microsoft Corporation, Redmond, WA), and data were extracted using the same program.

The statistical analysis was mainly designed to detect the diagnostic concordance (expressed as the percentage of matching diagnosis) in detection of AOM episodes between primary care/emergency room paediatricians and our internal validator (FF).

The results are given as absolute numbers and percentages, or arithmetical mean values ± standard deviation (SD). The data were analysed using STATA 10.0 software (StataCorp, College Station, TX, USA); a *p*-value of < 0.05 was considered statistically significant.

## Results

### General features of cases included in the study

One hundred and thirty-four single AOM episodes (65 on the right ear, 69 on the left ear) occurring in 87 children (mean age: 26.9 +/- 18.9 months) were included in the analysis (Table 1).

**Table 1:** Distribution of the demographic features of the included children

Age	n. (%)	Males (%)	Females (%)
Total	134 (100%)	71 (53%)	63 (47%)
0–6 months	3 (2.2%)	1 (33.3%)	2 (66.3%)
6–24 months	72 (53.7%)	40 (55.5%)	32 (45.5%)
24–60 months	48 (35.2%)	25 (52%)	23 (48%)
60–120 months	11 (8.2%)	5 (45%)	6 (55%)

Legend: N. = number

Ninety-nine out of 134 (73.9%) AOM episodes were diagnosed by primary care paediatricians in 65 children with a mean age of 56.7 +/- 8.1 months, and the remaining 35 AOM episodes (26.1%) by emergency room paediatricians in 22 children having a mean age of 46.9 +/- 9.8 months.

### Diagnostic concordance of AOM episodes

Ninety-seven out of 134 (72.4%) AOM diagnoses occurring in 62 children aged 26.8 +/- 18.9 months were validated by our internal validator; in all of these, the TM had been described as bulged and hyperaemic. Seventy-four out of 97 (76.3%) validated diagnoses had been performed by primary care paediatricians, and the remaining 23 (23.7%) by emergency room paediatricians. Acute onset of symptoms had been reported in all of 97 episodes; associated symptoms were: ear pain in 49 (50.5%) cases, fever in 51 (52.6%) cases, and both of them in 28 (28.9%) cases. The presence of non-impacting earwax was described in 2 (2.1%) cases, in which however a punctual description of the TM was provided.

**Fig. 1:**
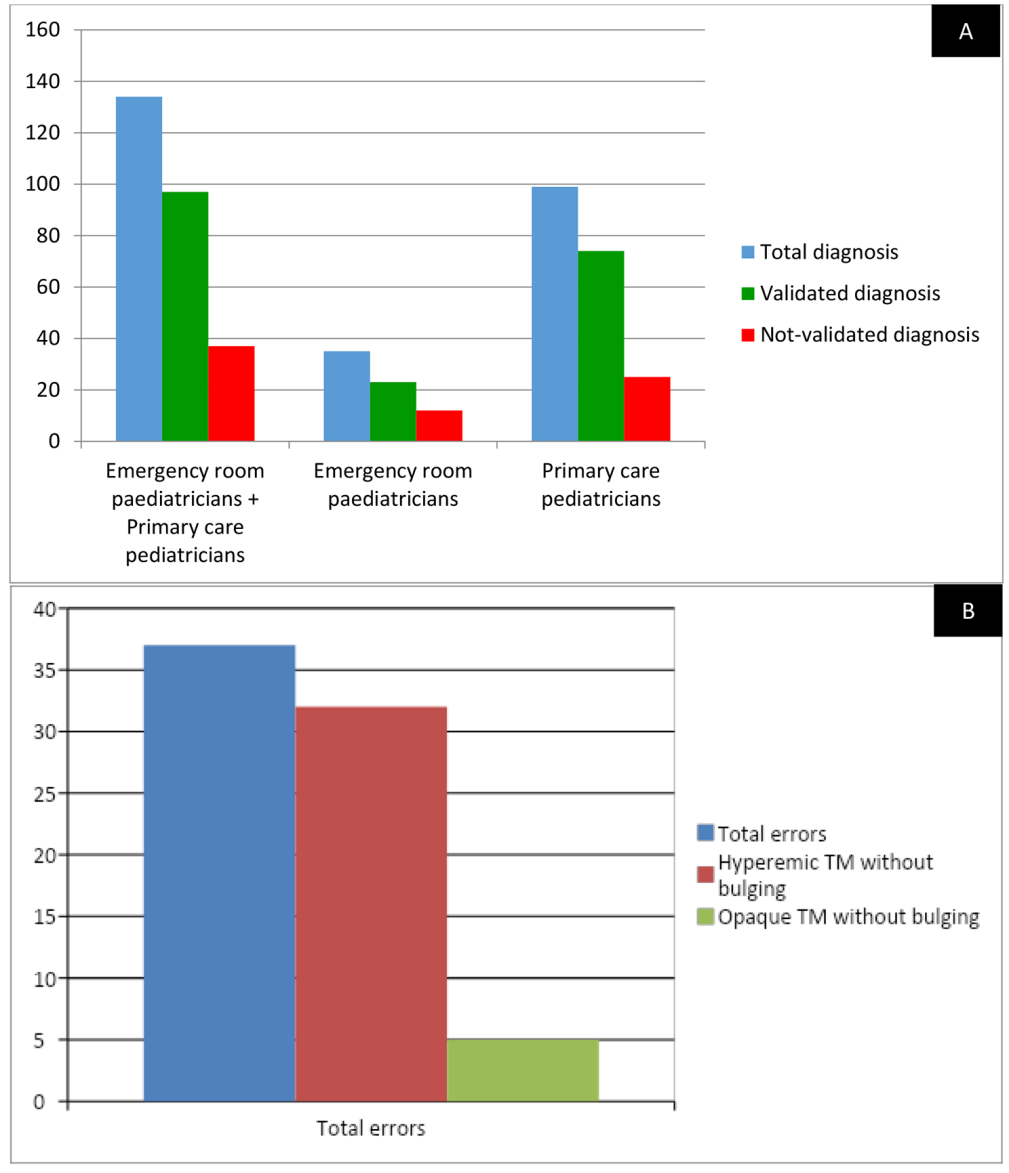
Distribution of the main diagnostic errors compared to the total number of non-validated diagnoses (**A**); and distribution of validated and non-validated diagnoses according to physicians (**B**)

Thirty-seven out of 134 (27.6%) AOM diagnoses occurring in 25 children aged 26.4 +/- 18.5 months were not validated. Twenty-five out of 37 (67.6%) not-validated diagnoses had been made by primary care paediatricians and the remaining 12 (32.4%) by emergency room paediatricians. In 32 out of 37 (86.5%) not-validated diagnoses, the TM had been described as hyperaemic without bulging; in the remaining 5 (15.0%) cases, it had been described only as opaque. Concerning the TM position, among these 37 not-validated diagnoses it had been reported as retracted in 5 (13.5%) cases, while bulging had never been identified. Symptoms reported among these 37 cases were: ear pain (21/37, 56.7%), fever (18/37, 48.6%), ear pain + fever (9/37, 24.3%); in 7/37 (18.9%) cases the children were asymptomatic (Fig. 1A and B).

Diagnostic concordance in detection of AOM episodes between primary care/emergency room paediatricians and our internal validator was 72.4%. Overall, in 25/99 (25.2%) cases primary care paediatricians and in 12/35 (34.2%) cases emergency room paediatricians concluded with a not confirmed diagnosis of AOM.

## Discussion

Our study points out that AOM diagnosis still represents a relevant issue for healthcare providers, with potential relevant consequences in terms of costs and unnecessary antibiotic treatments [10].

Our analysis showed that 27.6% of AOM diagnosis performed by paediatricians were not validated by our internal validator; this finding is in line with previous data, which hypothesized that AOM could be overdiagnosed in about 30% of children [11–13].

The most common diagnostic pitfall detected was the misleading diagnosis of AOM in case of hyperaemic TM without bulging (32/37); the following most common mistake was the misleading diagnosis in case of opaque TM without bulging (5/37). While performing otoscopy in case of suspected AOM, particular attention should be paid to the identification of a bulging eardrum, as this finding better correlates with a bacterial infection in the middle ear and represents the most consistent sign of AOM [14–16].

On the contrary, other otoscopic findings such as an isolated hyperaemia of the TM (frequent finding in the infant during crying), the loss of the bright triangle, the retraction of the TM and air-fluid levels should not be considered diagnostic criteria for AOM [1, 17, 18]. On the contrary, these otoscopic findings are in most cases suggestive for otitis media with effusion (OME), defined as the presence of middle ear fluid (MEE) without signs or symptoms of acute infection [19].

AOM misdiagnosis in case of OME represents one of the most common diagnostic pitfalls and still remains a frequent condition in which antibiotics are prescribed inappropriately [8, 18, 20]. An investigation by Shaikh et al. on 263 children documented how the bulging of the TM was identified in 92% of those with AOM, compared to 0% of those with OME; moreover, in the same paper the authors developed a diagnostic algorithm focused on the bulging of the TM as the starting point to discriminate between AOM and OME: if any bulging is not identified, AOM diagnosis should be set aside [21]. A more recent survey by Chiappini et al. was conducted in 2019 through a questionnaire administered to pediatricians, with a main focus on the therapeutic management of the disease: similarly to our results, only in 57.8% of cases a bulging TM was considered among the most relevant signs of AOM, while in 25.2% of cases a retraction of the eardrum was considered as a major sign [22].

Performing otoscopy can be challenging in younger children, especially in those who are scared and not cooperative; however, in our analysis, the age of the child did not influence the rate of error, as the mean age was almost equivalent in the group of correct diagnosis and in that of incorrect ones.

The rate of misdiagnosis was slightly higher among emergency room paediatricians than among primary care ones (34.2% vs. 25.2%). Compared to our results, an Australian retrospective cohort study reported lower rates of error and a good diagnostic accuracy in the emergency department, with about 13% of diagnoses classified as “unlikely”; however, it should be noted that the remainder 87% of cases included in this study was composed by those defined as “likely” (60%) plus those defined as “possible” (22%), hence the percentage of misdiagnosis could have been underestimated [23]. The slightly higher error rate in the emergency department could be at least in part related to the different setting, as children coming to hospital are often less cooperative, parents could be in distress and the physician is able to dedicate less time to the child and its family. Moreover, in our opinion, this finding could be also in part related to the mean younger age of healthcare providers in the emergency department. Previous studies reported how otoscopic skills relate to the examiner’s experience, showing significant differences between medical students, residents, paediatricians and experts [24, 25].

This issue is tightly connected to the scarce training towards otoscopy and paediatric ENT diseases in general in our country: otoscopy is indeed a learned clinical skill that requires a reference bank of “normal” otologic variations [20]. Accordingly, literature shows a certain grade of discordance in recognizing otologic conditions among medical students, trainees and practitioners [26, 27]. Unfortunately, as reported in a survey by Marchisio et al., most of the Italian paediatricians and ENTs do not receive adequate AOM education during medical school and residency, especially among pediatricians, while among ENT schools paediatric conditions are often considered a minor issue [7]. As shown by Paul et al., students who receive a specific paediatric otoscopy curriculum had significant improvements in their skills when compared those who received a routine learning program. It is therefore essential to implement training programs focused on this topic in our schools to improve diagnostic accuracy; moreover, it should be mentioned that acquired skills tend to diminish over time, emphasizing the need for a continued practice [28].

Concerning the diagnostic tools, pneumatic otoscopy is the optimal choice to identify AOM [18]; however, it requires a certain training and it is not always available in the office or in the emergency room and its use is still limited in common practice [7]. On the other hand, it should be noted that an AOM diagnosis is still possible in case of severe bulging of the eardrum associated with signs of inflammation [29]. Unfortunately, in our study we cannot provide data on the use of the pneumatic otoscope; however, in a recent Italian survey, only 9.6% of primary care paediatricians declared to use instrument [22]. Among other devices, tympanometry is surely helpful in improving diagnostic accuracy and is perceived as easy to learn and to interpret by paediatricians [30]; however, it is quite expensive and is prevalently used by ENTs, while paediatricians are resorting to this tool only in 3.9% of cases [22].

A point of strength of our study is the evaluation of AOM diagnostic matching through the direct analysis of eardrum description provided by paediatricians on clinical records, rather than the administration of a questionnaire, thus giving an accurate report on this topic. One limitation is related to the inclusion of data extracted only from the Milan area; moreover, the diagnostic concordance has been evaluated on reports, rather than with a direct evaluation of physicians’ skills.

## Conclusions

AOM diagnosis still represents a relevant issue among paediatricians in our country, even though two national guidelines have been published in 2009 [31] and 2019 [1].

Misdiagnosing a frequent disease such as AOM implies higher costs for families and society, higher appeal to medical visits and diagnostic examinations and, above all, unnecessary antibiotic prescription and consumption.

Clinicians dealing with children during their practice should be confident with the otoscopic signs that indicate AOM. Nevertheless, beyond theory, it is fundamental to educate students and residents on performing otoscopic examination and on the interpretation of the most common findings. Hopefully, in the next future the technologic development will provide new tools which can facilitate both our everyday practice and education, with the aim to reduce as much as possible the rate of incorrect diagnosis of a condition which is highly frequent, yet often not so familiar.

### Electronic supplementary material

Below is the link to the electronic supplementary material.

Supplementary Material 1

## Data Availability

Data available on request due to privacy/ethical restrictions.

## Abbreviations

AOM: Acute otitis media
MEE: Middle-ear effusion
TM: Tympanic membrane
SD: Standard deviation
ENT: Ear nose and throat
