# Supplementary material for: Acute otitis media diagnosis in childhood: still a problem in 2023?
Source: Ital J Pediatr. 2024 Jan 25;50:19. doi: 10.1186/s13052-024-01588-y (PMC10809769; doi:10.1186/s13052-024-01588-y)
Supplement: Supplementary file 1 — Supplementary Material 1 [file 13052_2024_1588_MOESM1_ESM.docx]

**Reviewer #1:**

The article necessitates enhancement within the method section by providing additional details. These details should encompass demographic data, the precise timing of both the initial and subsequent evaluations, as well as a comprehensive account of the therapeutic interventions administered prior to the second evaluation etc.

Furthermore, if my comprehension is accurate, it appears that acute otitis media predominantly manifested in the cohort of younger children, and there is a notable absence of any reference to older children within the study.

Is it possible to include the protocol number and the prroval date of the Ethics Committee?

**RISPOSTA 1**

Dear reviewer, thank you for your suggestions.

Our study consisted in a retrospective chart review of children regularly followed in our patient clinic. We extrapolated episodes diagnosed as AOM from these clinical records (if a punctual description of the eardrum was provided) and analyzed whether the otoscopic findings reported by pediatricians matched with an actual description of what should appear as an AOM. Therefore, considering the retrospective nature of our analysis, we did not perform any initial or subsequent evaluation, nor any kind of therapeutic intervention was part of our study.

Concerning the age issue: it is well known that younger children suffer from AOM more than older ones, so this finding is not surprising for us. However, the aim of our study was to report the error rate and the most common pitfalls in diagnosing AOM in children, while defining the incidence of the disease in different ages was not the purpose of our study.

Demographic data useful to our analysis have been provided in the results section; however, a table including a more accurate description of our population in terms of age and sex has been added.

—---------------

**REVIEWER #2:**

Dear Authors,

AOM is a common clinical conditon that may cause chronic problems in childhood. The most important problem is chronic ear diseases such as cholestatoma. So it is important to diagnose and treat the patients by physicians. It is obivious that medical education in both medical schools and residency is not enough. So, expert guided case by case practical education is necessary. It is clear that ENT clinicians are more experienced than other clinicians about AOM. They use pneumatic otoscope, close followup, and tympanometric investigation.

There are many problems in this submission.

1.Abstract section

line 20. The definition of RAOM is not mentioned before.

2.Methodology: Validation should be done by a senior otorhinolaryongologist. Because seroz otitis media both chronic and acute, chronic otitis media may be misdiagnosed by other physicians. Another issue is the condition of external ear canal. External otitis media, myringitis, serumen or any foreign body may cause misdiagnoses.

As the diagnostic tool for AOM, Pneumotic otoscopy is more suitable .

3.Results:There is no any graphic, table or figure which strengthten the findings.

I think there are many methodologic problems. My advice is to redesign and fix the deficient parts and prepare new submission.

Best regards.

**RISPOSTA 2**

Dear reviewer, thank you for your suggestions.

1. RAOM definition in the abstract has been fixed.
2. Methodology: the otitis media outpatient clinic, directed by Prof. Paola Marchisio, regularly follows children with RAOM and diseases of the upper airways who are sent for an accurate evaluation from general practitioners. About 12-15 first evaluations and 40-50 visits per week are performed, of which 90% of the patients suffer from recurrent ear diseases; moreover, these children are evaluated with pneumatic otoscopy and tympanometry. We therefore believe that physicians (pediatricians in this case) who daily evaluate children suffering from RAOM and are perfectly skilled in pneumatic otoscopy are also absolutely capable in validating AOM diagnosis.
3. Tables and graphics have been provided

—-------------

**Reviewer #3**: Title and abstract:

The title is consistent with the presented problem and reflects the main message of the study.

Introduction:

The purpose of the article is clearly presented.

Material and methods:

There is no any ethical concerns about this study.

Research design is appropriate and methods are clearly explained.

Inclusion and exclusion criteria are clearly explained.

Essential characteristics of the sample is adequatly described and sample size is adequate and representative.

Data have been collected in systematic and comprenhensive manner.

Statistical methodology is appropriate.

Statistical analysis:

Statistical analysis is appropriate.

Results:

Analysis of the data is systematic.

Results are credible and important.

Discussion:

Interpretation of the results is clearly presented and adequately supported by the evidence adduced.

Conclusions:

Conclusions are logically valid and justified by the evidence adduced.

Tables and graphics:

All figures and tables are adequate and necessary.

References:

References are up-to- date and the most important studies have been cited.

General comments to the authors:

Manuscript is well prepared in term of content.

The results of the study are convincing.

The work deserves to be published.

Overall publication value:

The topic is important.

Citation potential is medium.

**NESSUNA RISPOSTA**
